# Addendum: Cellular differentiation into hyphae and spores in halophilic archaea

**DOI:** 10.1038/s41467-024-44821-2

**Published:** 2024-02-01

**Authors:** Shu-Kun Tang, Xiao-Yang Zhi, Yao Zhang, Kira S. Makarova, Bing-Bing Liu, Guo-Song Zheng, Zhen-Peng Zhang, Hua-Jun Zheng, Yuri I. Wolf, Yu-Rong Zhao, Song-Hao Jiang, Xi-Ming Chen, En-Yuan Li, Tao Zhang, Pei-Ru Chen, Yu-Zhou Feng, Ming-Xian Xiang, Zhi-Qian Lin, Jia-Hui Shi, Cheng Chang, Xue Zhang, Rui Li, Kai Lou, Yun Wang, Lei Chang, Min Yin, Ling-Ling Yang, Hui-Ying Gao, Zhong-Kai Zhang, Tian-Shen Tao, Tong-Wei Guan, Fu-Chu He, Yin-Hua Lu, Heng-Lin Cui, Eugene V. Koonin, Guo-Ping Zhao, Ping Xu

**Affiliations:** 1 Yunnan Institute of Microbiology, Key Laboratory for Microbial Resources of the Ministry of Education, School of Life Sciences, Yunnan University, Kunming, 650091 China; 2 State Key Laboratory of Proteomics, Beijing Proteome Research Center, National Center for Protein Sciences Beijing, Research Unit of Proteomics & Research and Development of New Drug，Research Unit of Proteomics Driven Cancer Precision Medicine, Chinese Academy of Medical Sciences, Institute of Lifeomics, Beijing, 102206 China; 3 National Center for Biotechnology Information, National Library of Medicine, National Institutes of Health, 8600 Rockville Pike, Bethesda, MD 20894 USA; 4 Henan Key Laboratory of Industrial Microbial Resources and Fermentation Technology, College of Biological and Chemical Engineering, Nanyang Institute of Technology, Nanyang, 473004 China; 5 College of Life Sciences, Shanghai Normal University, Shanghai, 200234 China; 6 Shanghai-MOST Key Laboratory of Health and Disease Genomics, Chinese National Human Genome Center at Shanghai and Shanghai Institute for Biomedical and Pharmaceutical Technologies, Shanghai, 201203 China; 7 Hebei Province Key Lab of Research and Application on Microbial Diversity, College of Life Sciences, Hebei University, Hebei, 071002 China; 8 Key Laboratory of Desert and Desertification, Northwest Institute of Eco-Environment and Resources, Chinese Academy of Sciences, Lanzhou, 730000 China; 9 Xinjiang Institute of Microbiology, Xinjiang Academy of Agricultural Science, Urumqi, 830091 China; 10 Biotechnology and Genetic Germplasm Resources Research Institute, Yunnan Academy of Agricultural Sciences, Kunming, 650205 China; 11 Department of Microbiology, Key Laboratory of Combinatorial Biosynthesis and Drug Discovery of Ministry of Education, School of Pharmaceutical Sciences, Wuhan University, Wuhan, 430072 China; 12 College of Food and Biological Engineering, Xihua University, Chengdu, 610039 China; 13 School of Food and Biological Engineering, Jiangsu University, Zhenjiang, 212013 China; 14 Key Laboratory of Medical Molecular Virology (MOE/NHC/CAMS), School of Basic Medical Sciences and Department of Microbiology and Microbial Engineering, School of Life Sciences, Fudan University, Shanghai, 200032 China; 15 Guizhou University, School of Medicine, Guiyang, 550025 China; 16 State Key Laboratory of Dampness Syndrome of Chinese Medicine, The Second Affiliated Hospital of Guangzhou University of Chinese Medicine, Guangzhou, 510120 China

**Keywords:** Archaeal biology, Taxonomy, Archaeal evolution, Cellular microbiology

Addendum to: *Nature Communications* 10.1038/s41467-023-37389-w, published online 01 April 2023

In this Article, we described strain YIM 93972 as representative of a new species in a new archaeal genus for which the name *Actinoarchaeum* was proposed.

We would like to make readers aware that after publication of this Article, it was brought to our attention that the name *Actinoarchaeum* was not correctly formed according to the rules of the International Code of Nomenclature of Prokaryotes. The correct name of the genus, *Actinarchaeum*, has now been validated^1^.

In addition, we wish to clarify that Professor Aharon Oren (mentioned in the Acknowledgements section of this Article) did not propose the incorrect name *Actinoarchaeum*, but the correct one, *Actinarchaeum*.

## References

[1] Oren, A. & Göker, M. Validation List no. 215. Valid publication of new names and new combinations effectively published outside the IJSEM. *Int. J. Syst. Evol. Microbiol.*10.1099/ijsem.0.006173 (2024). 10.1099/ijsem.0.006173 38299482

