# Supplementary material for: Cellular differentiation into hyphae and spores in halophilic archaea
Source: Nat Commun. 2023 Apr 1;14:1827. doi: 10.1038/s41467-023-37389-w (PMC10067837; doi:10.1038/s41467-023-37389-w)
Supplement: Supplementary file 1 — Supplementary Information [file 41467_2023_37389_MOESM1_ESM.pdf]

Supplemental Figures

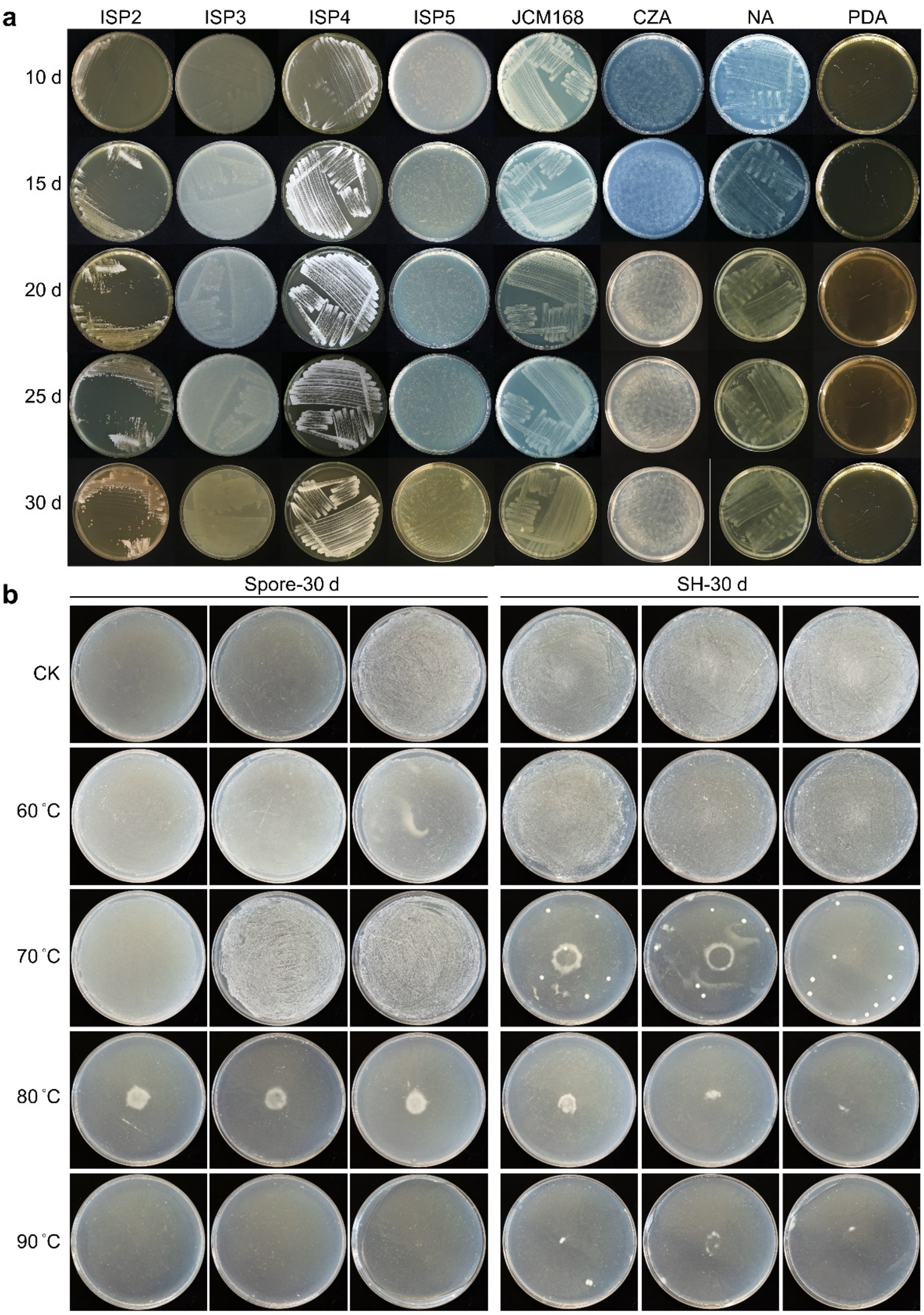

Supplementary Fig.1 | The characterization of strain YIM 93972. a, The phenotype comparison of strain YIM 93972 cultured on eight types of solid media (*n* = 3). ISP 2 (International Streptomyces Project 2, g.L^{-1}): yeast extract, 4; malt extract, 10; dextrose,

4; NaCl, 250; agar, 20, pH 7.2. ISP 3 (g.L^{-1}): oatmeal, 20; NaCl, 250; agar, 20; 1 mL trace salts solution (1% $FeSO_4 \cdot 7H_2O$, 1% $MnCl_2 \cdot 4H_2O$, 1% $ZnSO_4 \cdot 7H_2O$), pH 7.2. ISP 4 (g.L^{-1}): soluble starch, 10; K_2HPO_4, 1; MgSO4·$7H_2O$,1; $(NH_4)_2SO_4$, 2; $CaCO_3$, 2; NaCl, 250; agar, 20; 1 mL trace salts solution, pH 7.2. ISP 5 (g.L^{-1}): L-asparagine, 1; Glycerol, 10; K_2HPO_4, 1; NaCl, 250; agar, 20; 1 mL trace salts solution, pH 7.2. Nutrient Agar (NA) (g.L^{-1}): beef extract, 3; peptone, 5; NaCl, 250; agar, 20, pH 7.2. Czapek-Dox Agar (CZA) (g.L^{-1}): saccharose, 30; $NaNO_3$, 3; K_2HPO_4, 1; $MgSO_4$, 0.5; KCl, 0.5; $FeSO_4$, 0.01; NaCl, 250; agar, 20, pH 7.2. Potato Dextrose Agar (PDA) (g.L^{-1}): potato starch, 4; dextrose, 20; NaCl, 250; agar, 20, pH 7.2. JCM 168 (g.L^{-1}): casamino acid, 5; yeast extract, 5; sodium glutamate, 3; sodium citrate, 3; $MgSO_4$, 20; KCl, 2; NaCl, 250; agar, 20; 1 mL trace salts solution, pH 7.2. **b**, The phenotype comparison of spore and substrate hyphae (SH) under different heat stress conditions (*n*=3).

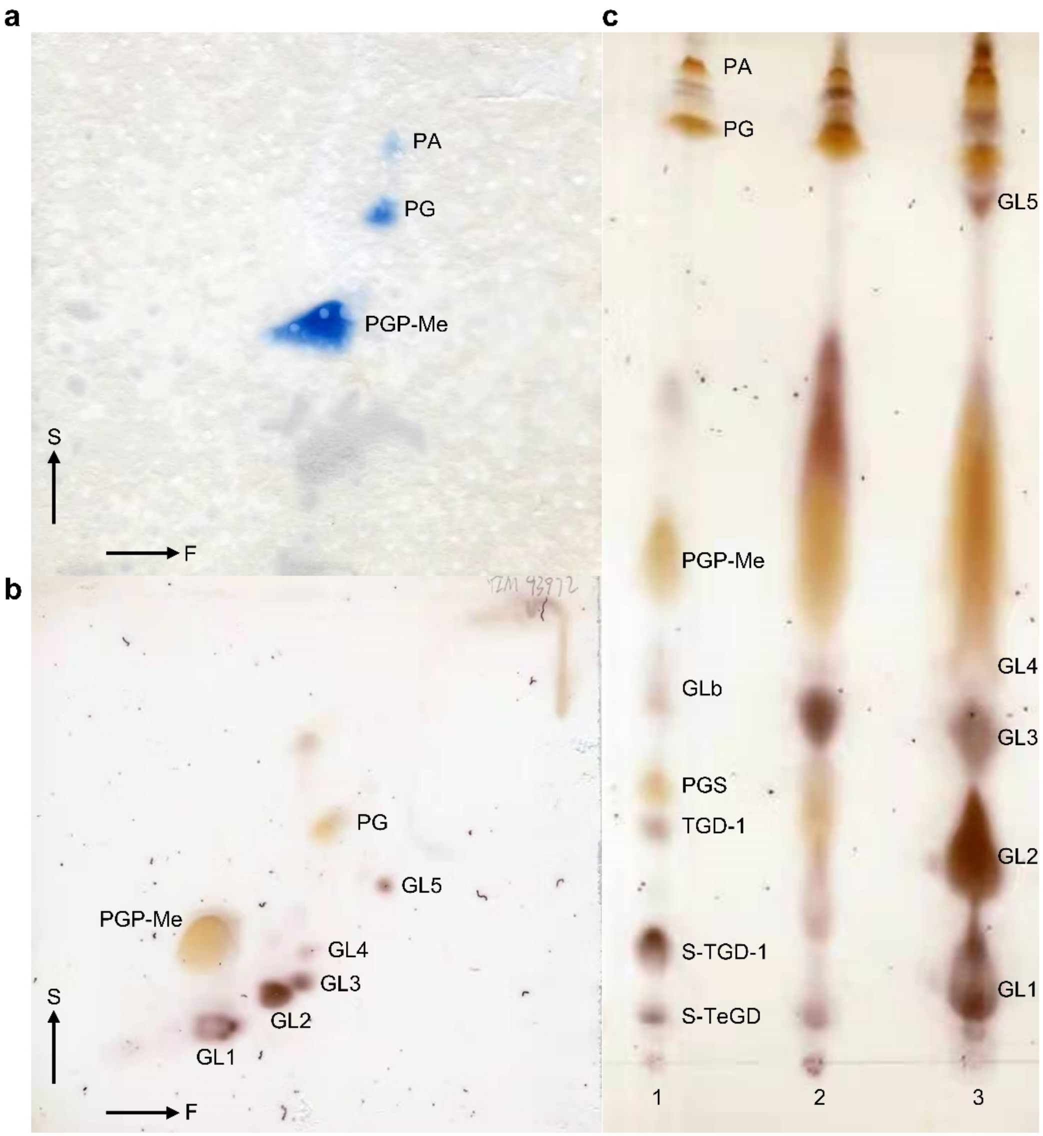

Supplementary Fig. 2 | Analysis of the polar lipid composition of strain YIM 93972 using thin layer chromatography. **a**, Two-dimensional TLC of the phospholipids from strain YIM 93972. **b**, Two-dimensional TLC of the phospholipids and glycolipids from strain YIM 93972. **c**, One-dimensional TLC of the phospholipids and glycolipids from strain YIM 93972 and related members. Lanes: 1, *Halobacterium salinarum* NRC 34001^T; 2, *Halomarina oriensis* JCM 16495^T; 3, YIM 93972. Spots are minor polar lipids of haloarchaeal strains tested. Abbreviations: GL, glycolipid; PA, phosphatidic acid; PG, phosphatidylglycerol; PGP-Me, phosphatidylglycerol phosphate methyl ester; S-TeGD, sulfated galactosyl mannosyl galactofuranosyl glucosyl diether; S-TGD-1, sulfated

galactosyl mannosyl glucosyl diether; TGD-1, galactosyl mannosyl glucosyl diether; F, first dimension of TLC; S, second dimension of TLC.

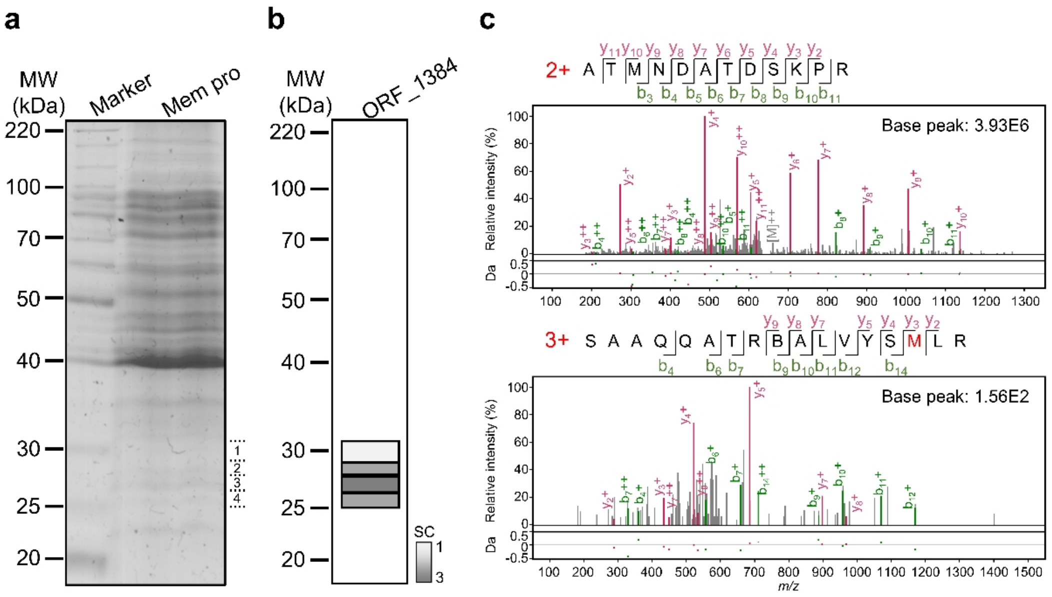

Supplementary Fig. 3 | Identification of halorhodopsin from YIM 93972 (*n*=3). a, Separation of membrane proteins by a 10% SDS-PAGE. **b**, The in silico reconstructed Western blots of the protein ORF_1384. The color was used to represent the number and distribution of spectral counts by LC-MS. **c**, The identified spectra for ORF_1384.

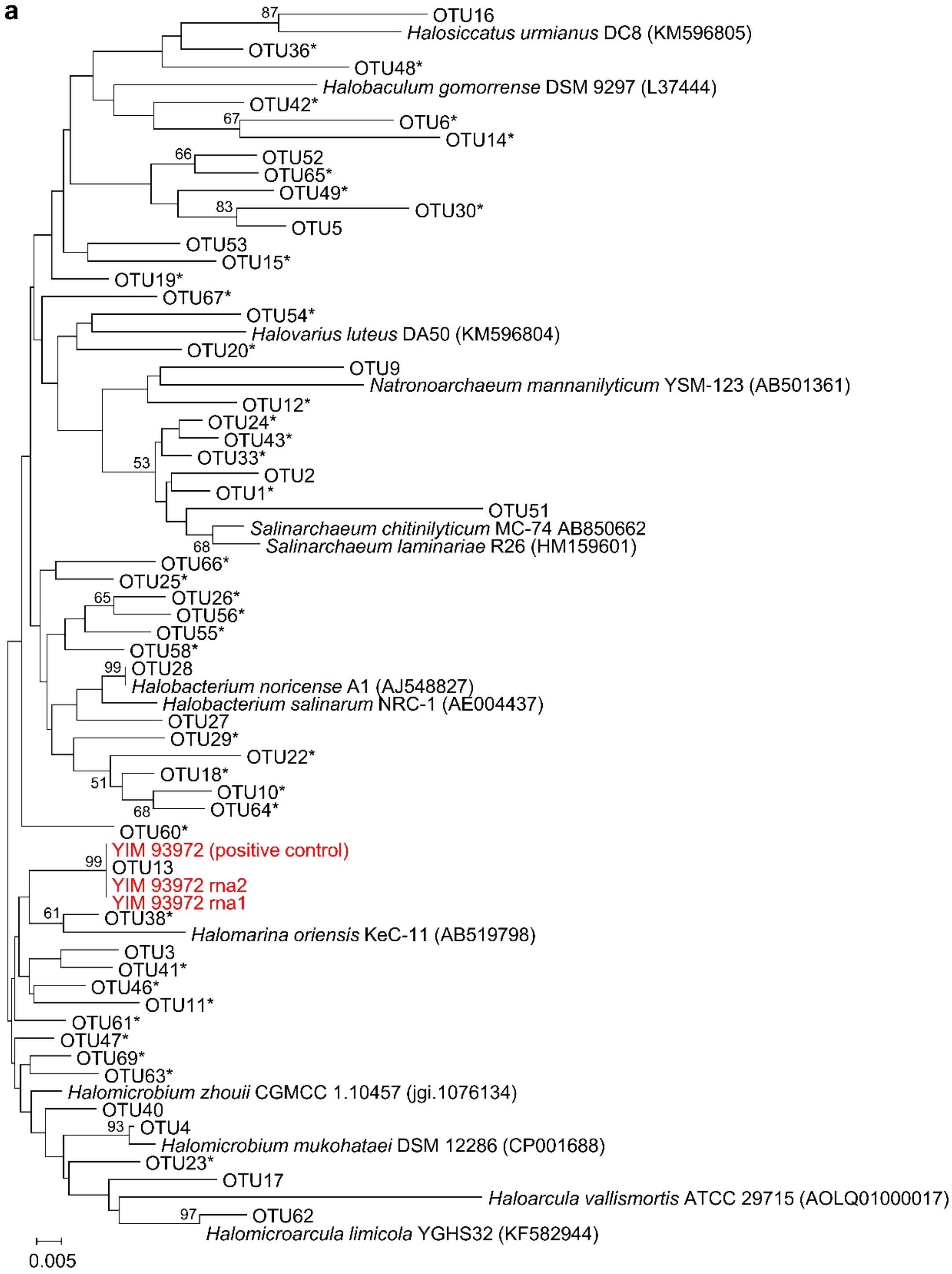
a
OTU16
Halosiccatus urmianus DC8 (KM596805)
OTU36*
OTU48*
Halobaculum gomorrense DSM 9297 (L37444)
OTU42*
OTU6*
OTU14*
OTU52
OTU65*
OTU49*
OTU30*
OTU5
OTU53
OTU15*
OTU19*
OTU67*
OTU54*
Halovarius luteus DA50 (KM596804)
OTU20*
OTU9
Natronoarchaeum mannanilyticum YSM-123 (AB501361)
OTU12*
OTU24*
OTU43*
OTU33*
OTU2
OTU1*
OTU51
Salinarchaeum chitinilyticum MC-74 AB850662
Salinarchaeum laminariae R26 (HM159601)
OTU66*
OTU25*
OTU26*
OTU56*
OTU55*
OTU58*
OTU28
Halobacterium noricense A1 (AJ548827)
Halobacterium salinarum NRC-1 (AE004437)
OTU27
OTU29*
OTU22*
OTU18*
OTU10*
OTU64*
OTU60*
YIM 93972 (positive control)
OTU13
YIM 93972 rna2
YIM 93972 rna1
OTU38*
Halomarina oriensis KeC-11 (AB519798)
OTU3
OTU41*
OTU46*
OTU11*
OTU61*
OTU47*
OTU69*
OTU63*
Halomicrobium zhouii CGMCC 1.10457 (jgi.1076134)
OTU40
OTU4
Halomicrobium mukohataei DSM 12286 (CP001688)
OTU23*
OTU17
Haloarcula vallismortis ATCC 29715 (AOLQ01000017)
OTU62
Halomicroarcula limicola YGHS32 (KF582944)
0.005

b

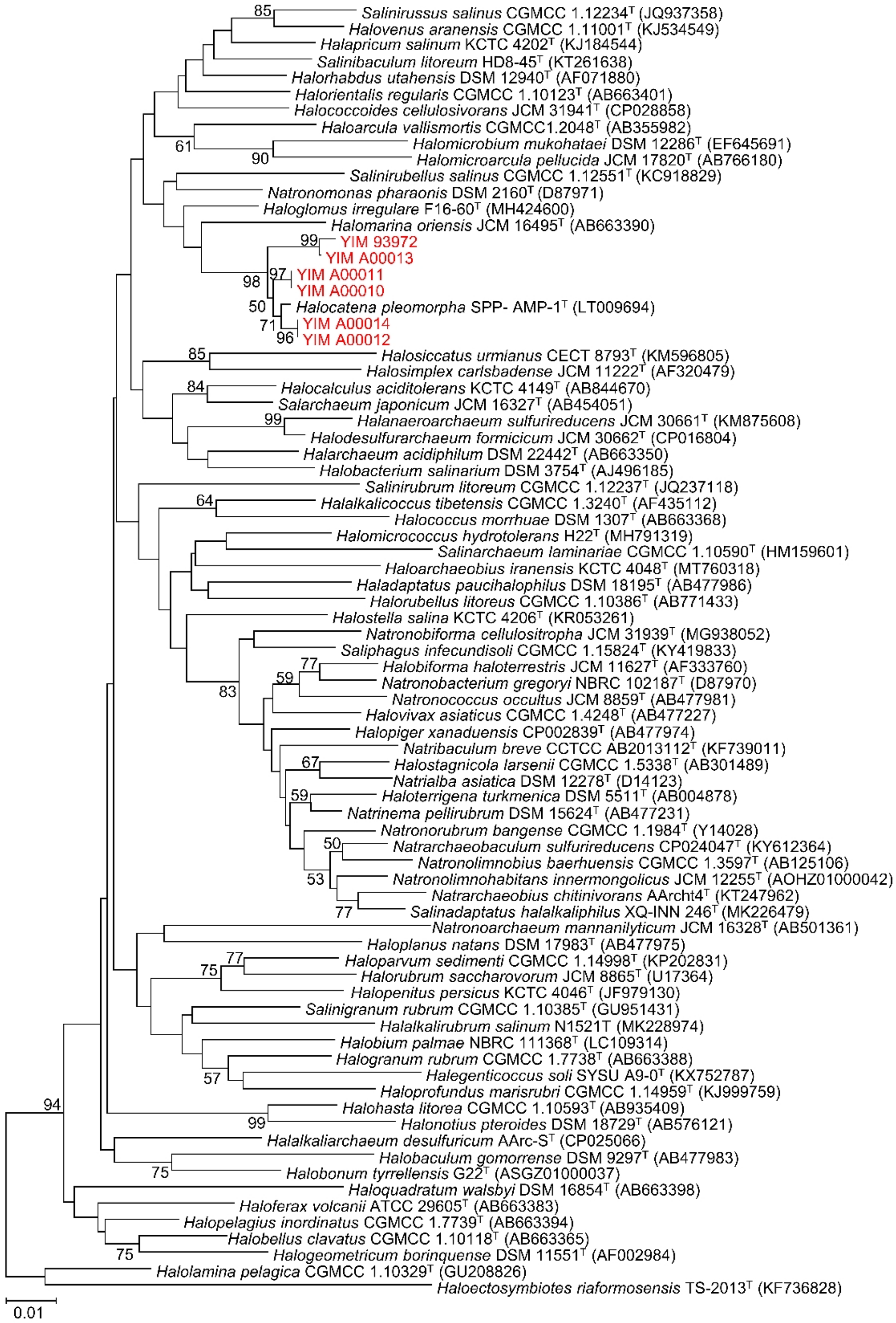

Supplementary Fig. 4 | Phylogenetic tree of 16S rRNA gene. a, Environmental SSU rRNA and rRNA sequence comparison. A phylogenetic tree (NJ) based on SSU rRNA and rRNA sequence of two Aiding Lake environmental samples. Percentage bootstrap values >50% are given at branch points. GenBank accession numbers of the type strains of *Halobacteria* are given in parentheses. Bar, 0.05 substitutions per nucleotide

position; Bootstrap value, 1000. *, uncultured species. (note: sample1:38, Aiding lake sample1:38 sequences; sample2:9, Aiding lake sample2: 9 sequences.) **b**, Phylogenetic tree of six novel strains with morphological differentiation and closest relatives based on 16S rRNA genes by the neighbour-joining method.

a

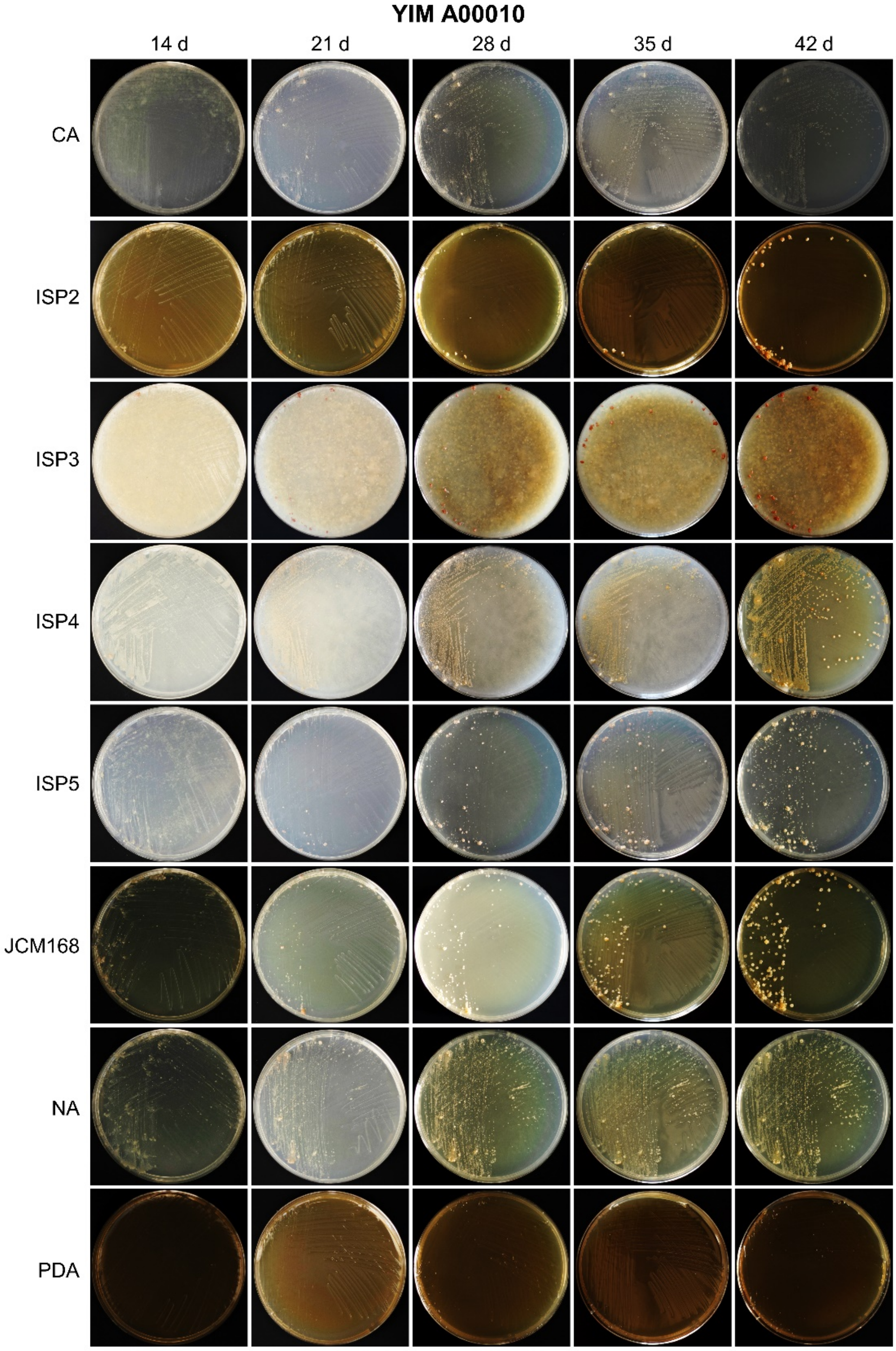

YIM A00010
14 d
21 d
28 d
35 d
42 d
CA
ISP2
ISP3
ISP4
ISP5
JCM168
NA
PDA

b

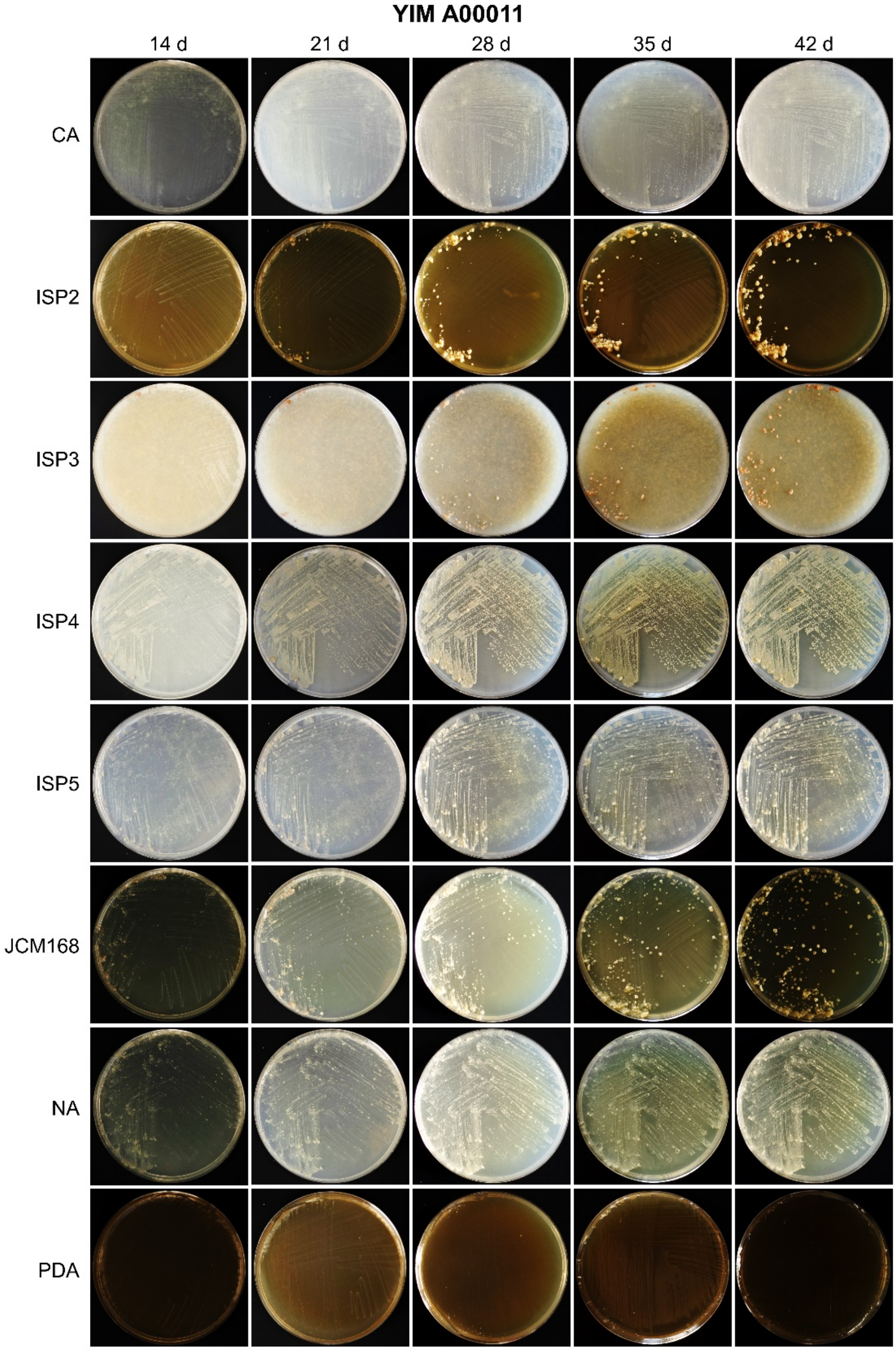
YIM A00011
14 d
21 d
28 d
35 d
42 d
CA
ISP2
ISP3
ISP4
ISP5
JCM168
NA
PDA

c

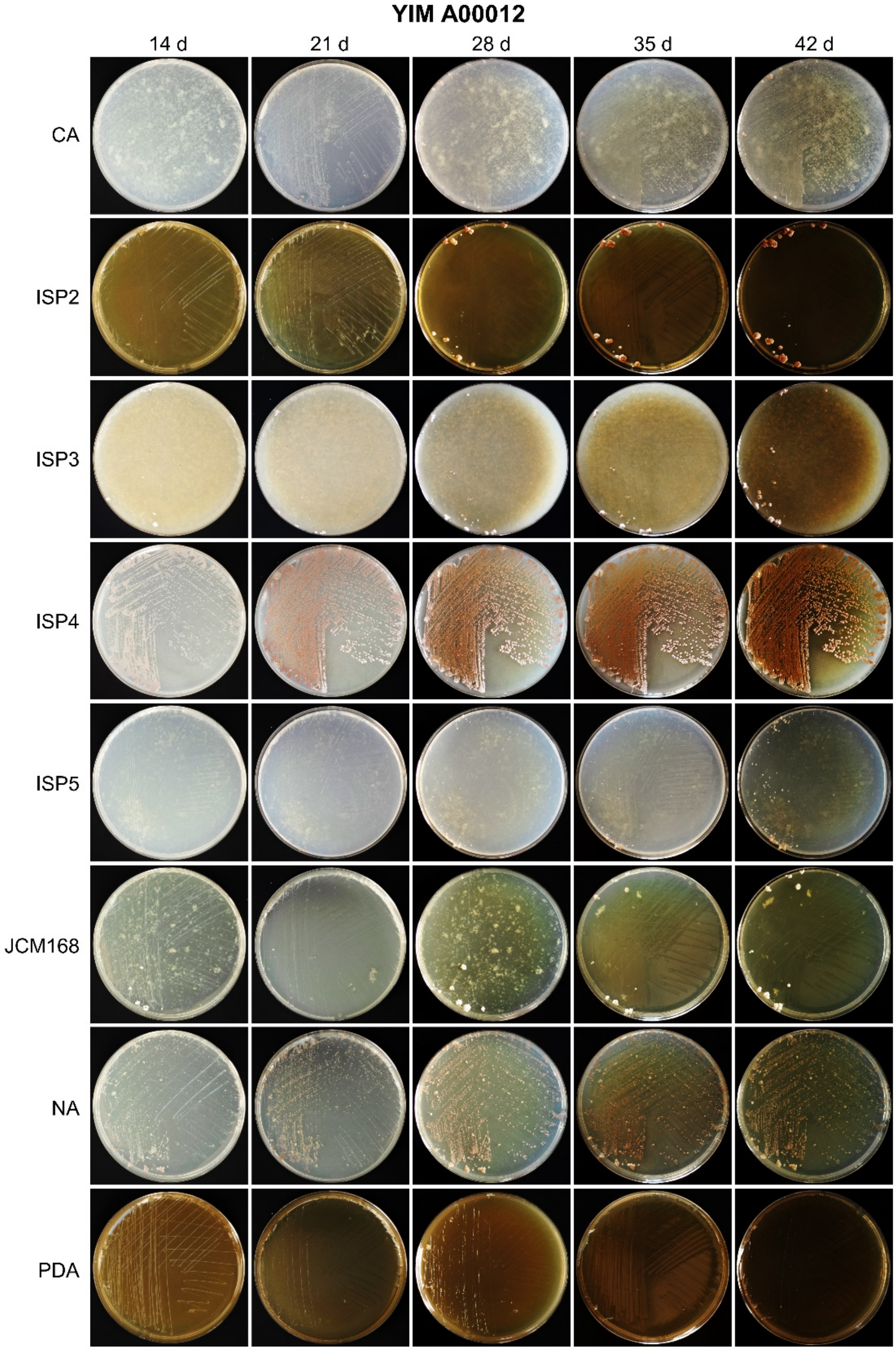
YIM A00012
14 d
21 d
28 d
35 d
42 d
CA
ISP2
ISP3
ISP4
ISP5
JCM168
NA
PDA

d

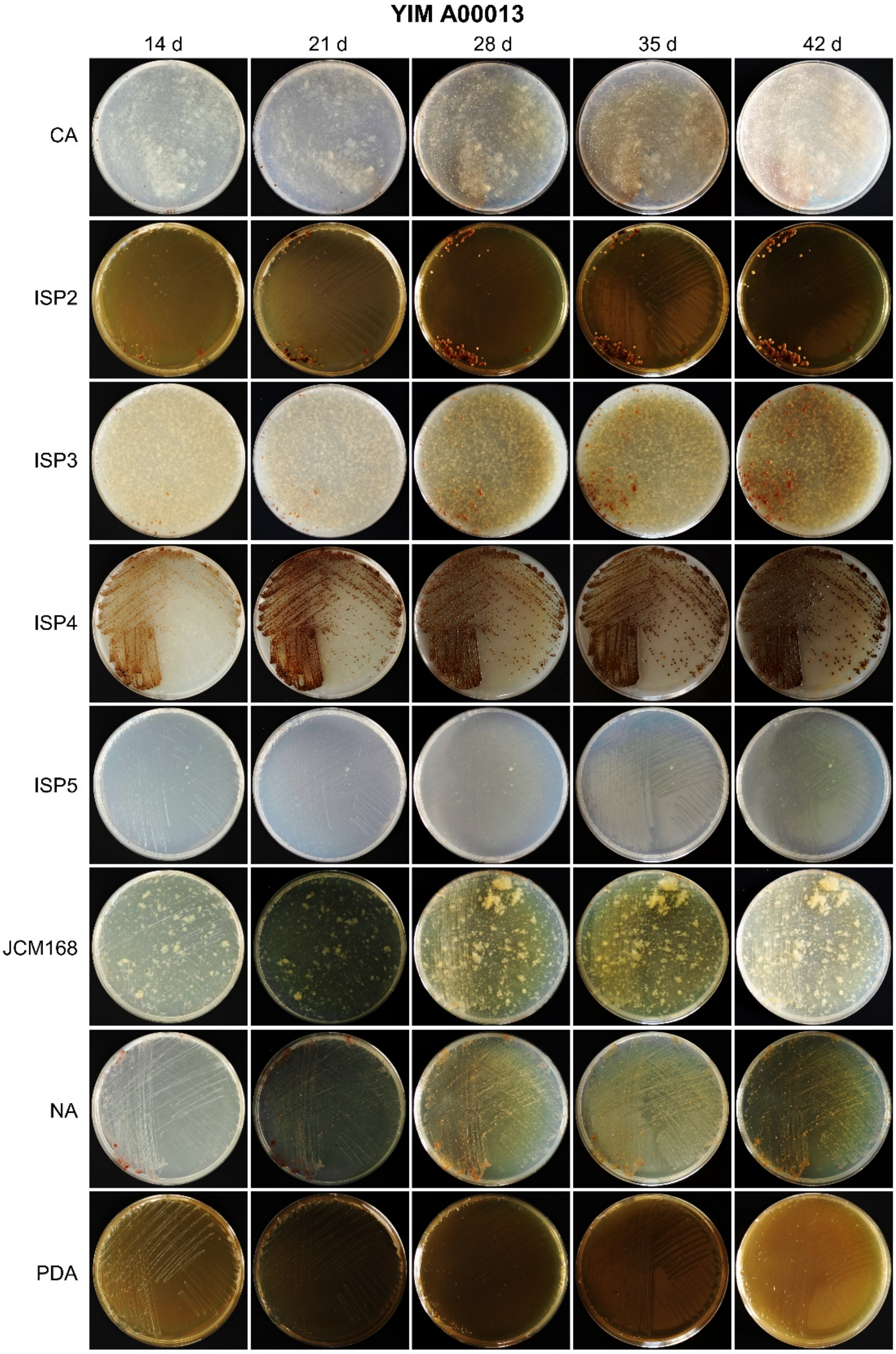
YIM A00013
14 d
21 d
28 d
35 d
42 d
CA
ISP2
ISP3
ISP4
ISP5
JCM168
NA
PDA

e

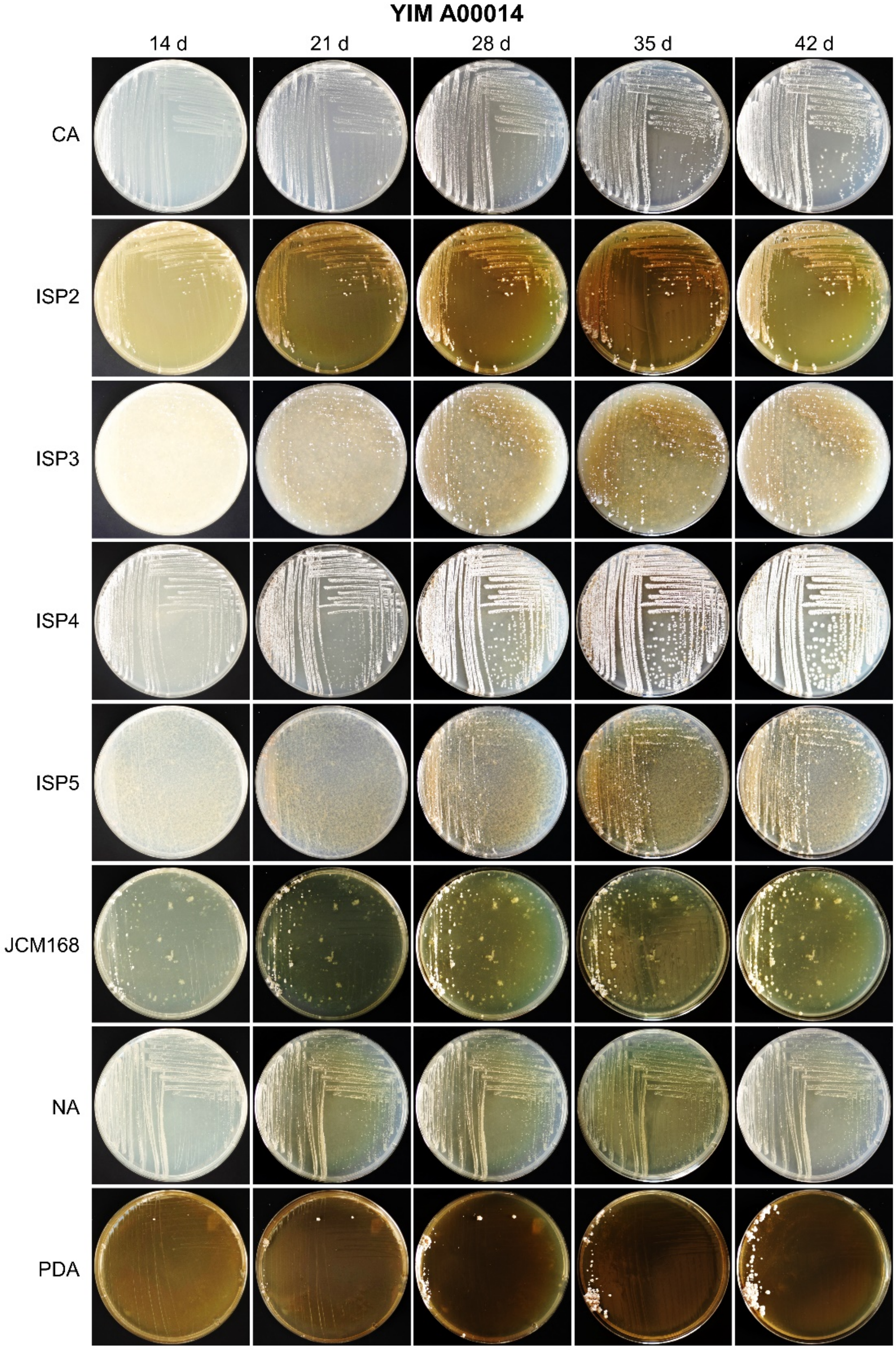
YIM A00014
14 d
21 d
28 d
35 d
42 d
CA
ISP2
ISP3
ISP4
ISP5
JCM168
NA
PDA

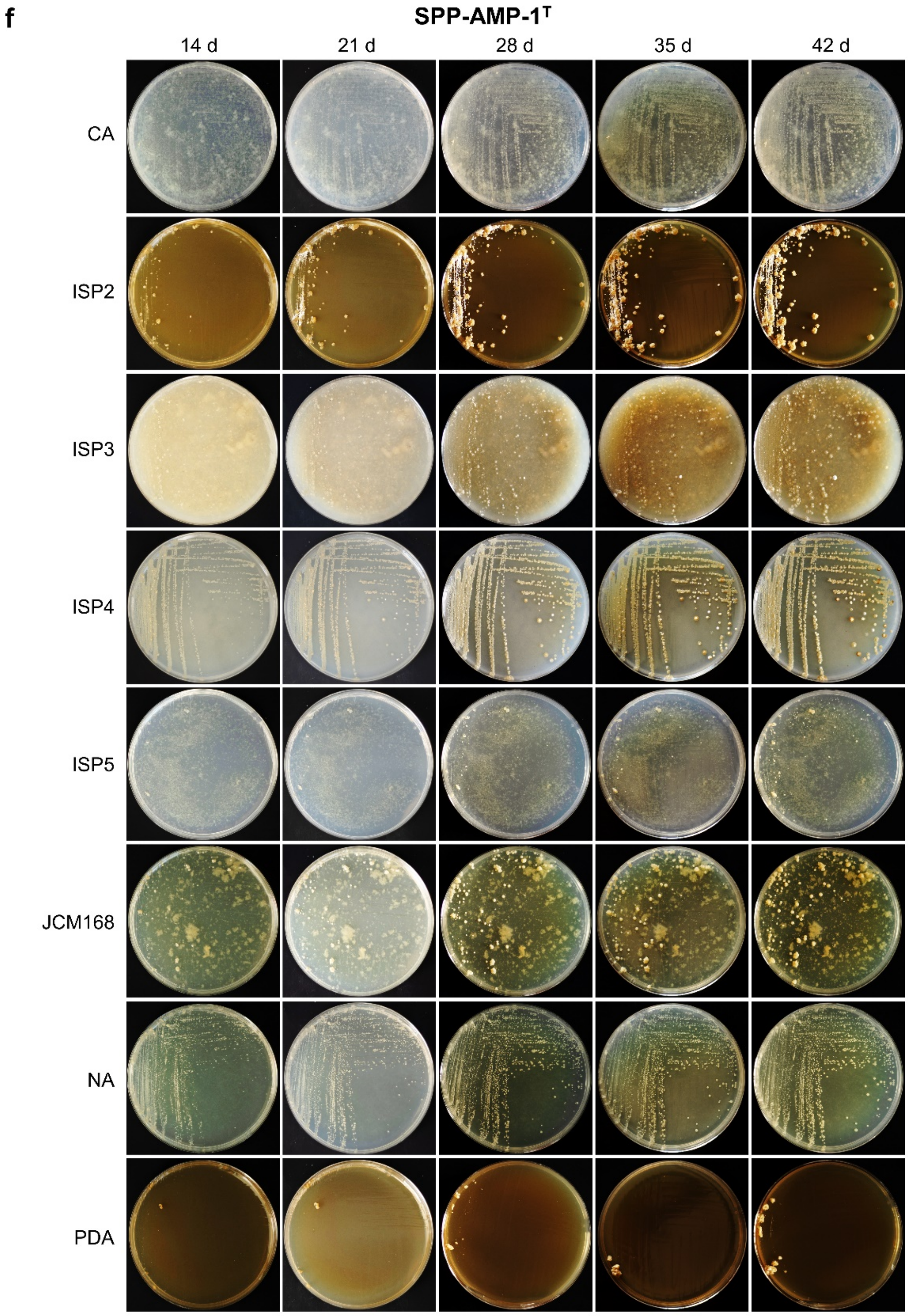

Supplementary Fig. 5 |The characterization of other six cellular differentiational strains (*n* = 3). a, YIM A00010. **b**, YIM A00011. **c**, YIM A00012. **d**, YIM A00013. **e**, YIM A00014. **f**, SPP-AMP-1^{T}.

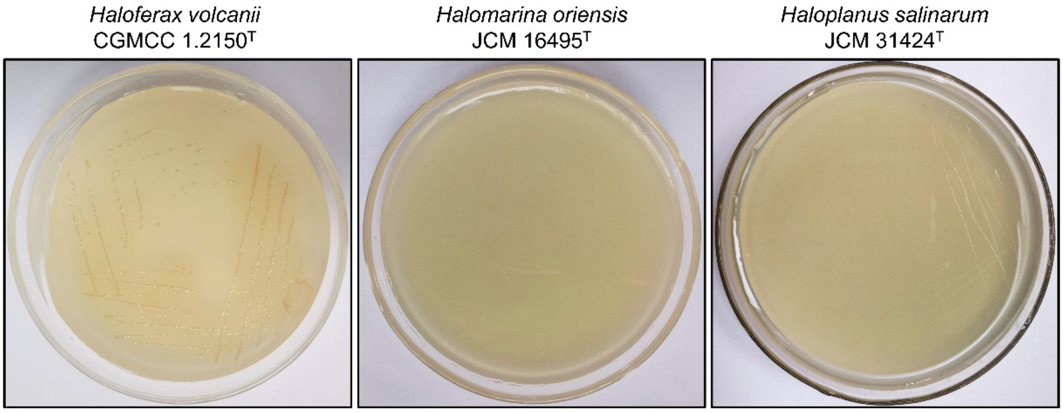

Supplementary Fig. 6 | The characterization of typical halophilic archaea on ISP 4 medium (*n* = 3).

a **Complete genome sequence of strain YIM 93972.**

Component of chromosome	Property
No. of contig	5
Total size	3,794,817 bp
Size of chromosome 1	2,676,592 bp
Size of chromosome 2	844,905 bp
Size of plasmid 1	99,700 bp
Size of plasmid 2	84,896 bp
Size of plasmid 3	41,997 bp
G+C content	56.31%
Coding sequences	3744
Consensus concordance	99.99%
Ribosomal RNAs	2 x (16S-23S-5S)
Transfer RNAs	47

b

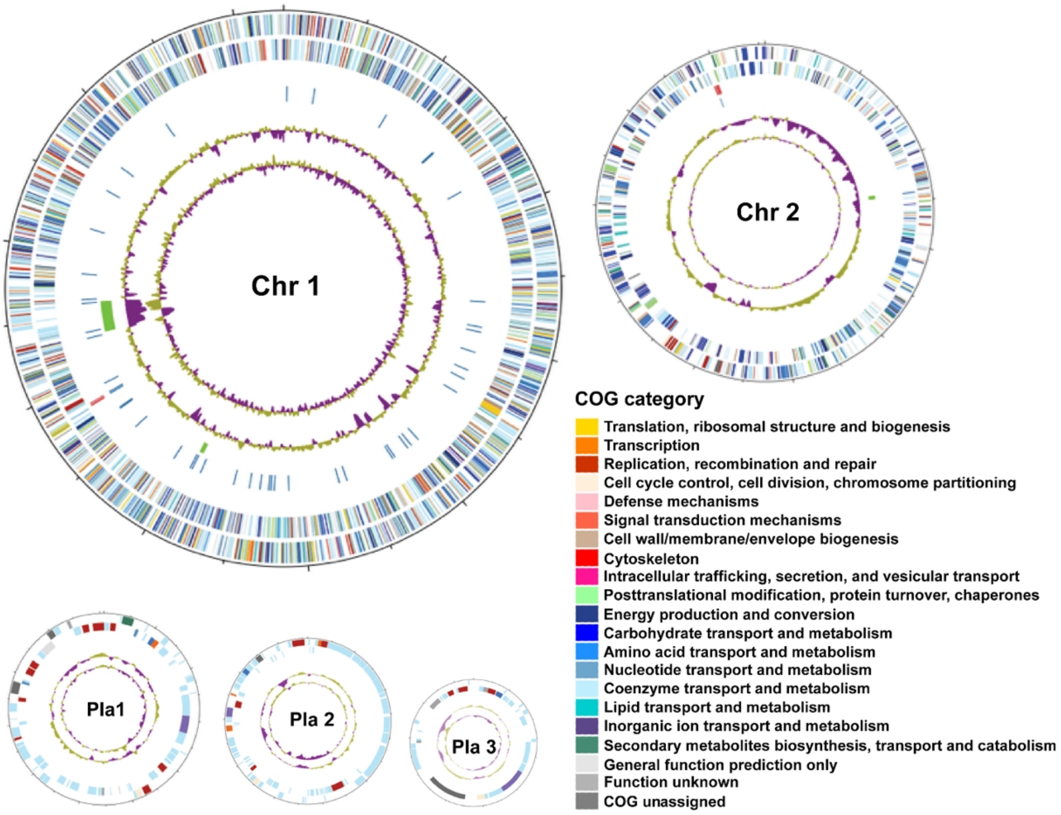

Supplementary Fig. 7 | The whole genome features of strain YIM 93972. a, The detail information of genome sequencing. **b**, Circular representation of the genome of YIM 93972. The whole genome consists of two chromosomes and three plasmids. Circles 1 and 2 (from the outside in), all genes (reverse and forward strand, respectively) colour-coded by COG function; circle 3, selected 'essential' genes (for replication, recombination and repair

genes); circle 4, tRNA; circle 5, rRNA; circle 6, G + C content; circle 7, GC bias [(G - C/G + C), khaki indicates values >1, purple < 1].

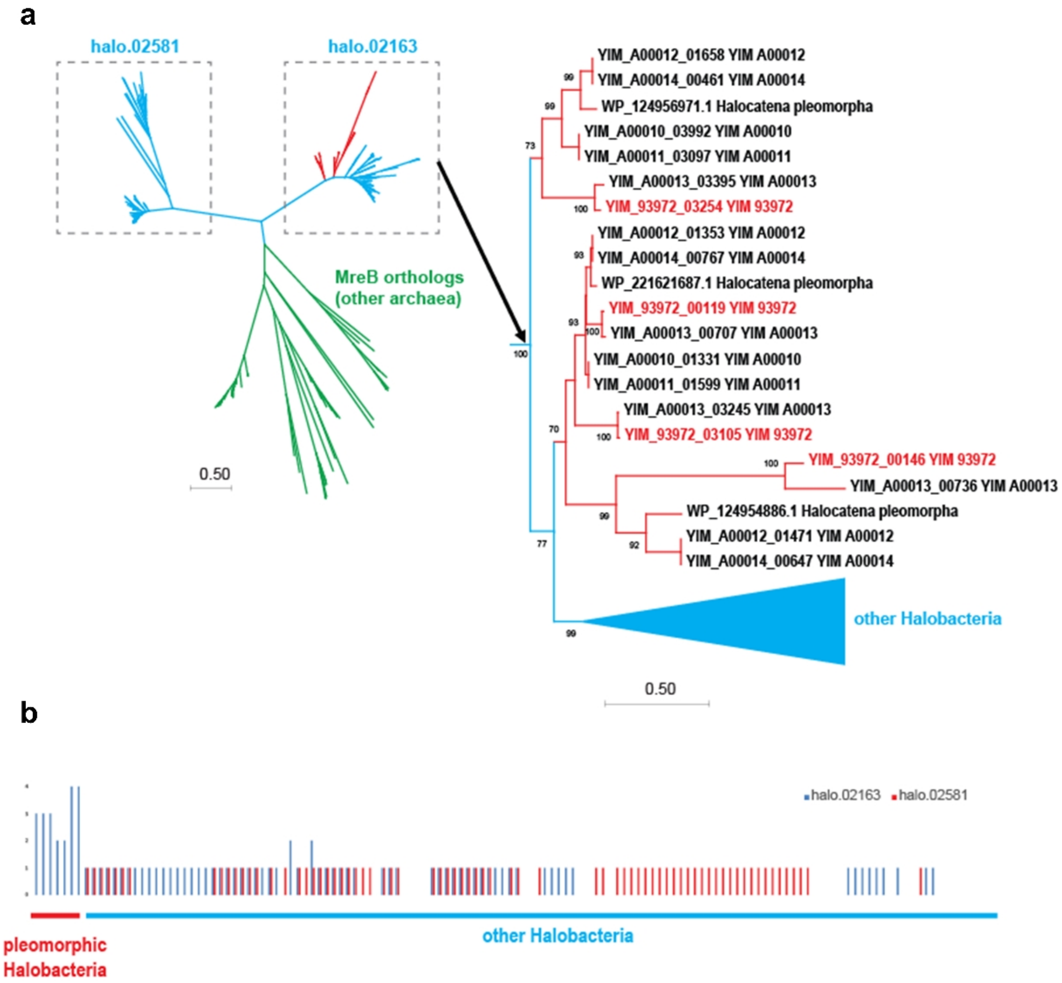

Supplementary Fig. 8 | MreB-like protein family expansion in *Halobacteria* form morphogenetic group. a, Phylogenetic tree of MreB-like protein family in archaea. The tree was built using FastTree program (WAG evolutionary model, gamma-distributed site rates) and includes all full-size halobacterial proteins from haloCOGs halo.02581 and halo.02163 and those from other archaea in arCOG03061 and arCOG03062; Blue – *Halobacteria*; Red – morphogenetic group; Green - other archaea. The subtree for halo.02163 is shown on the right. Sequences from YIM 93972 are highlighted by red. The support values are calculated using Fasttree program and shown for each bifurcation. The Scale is in substitutions for amino acid site. **b,** Presence/absence and the number of paralogs of halo.02581 and halo.02163 in morphogenetic and other *Halobacteria*. Number of paralogs is on the Y-axis. Data is shown for 130 genomes.

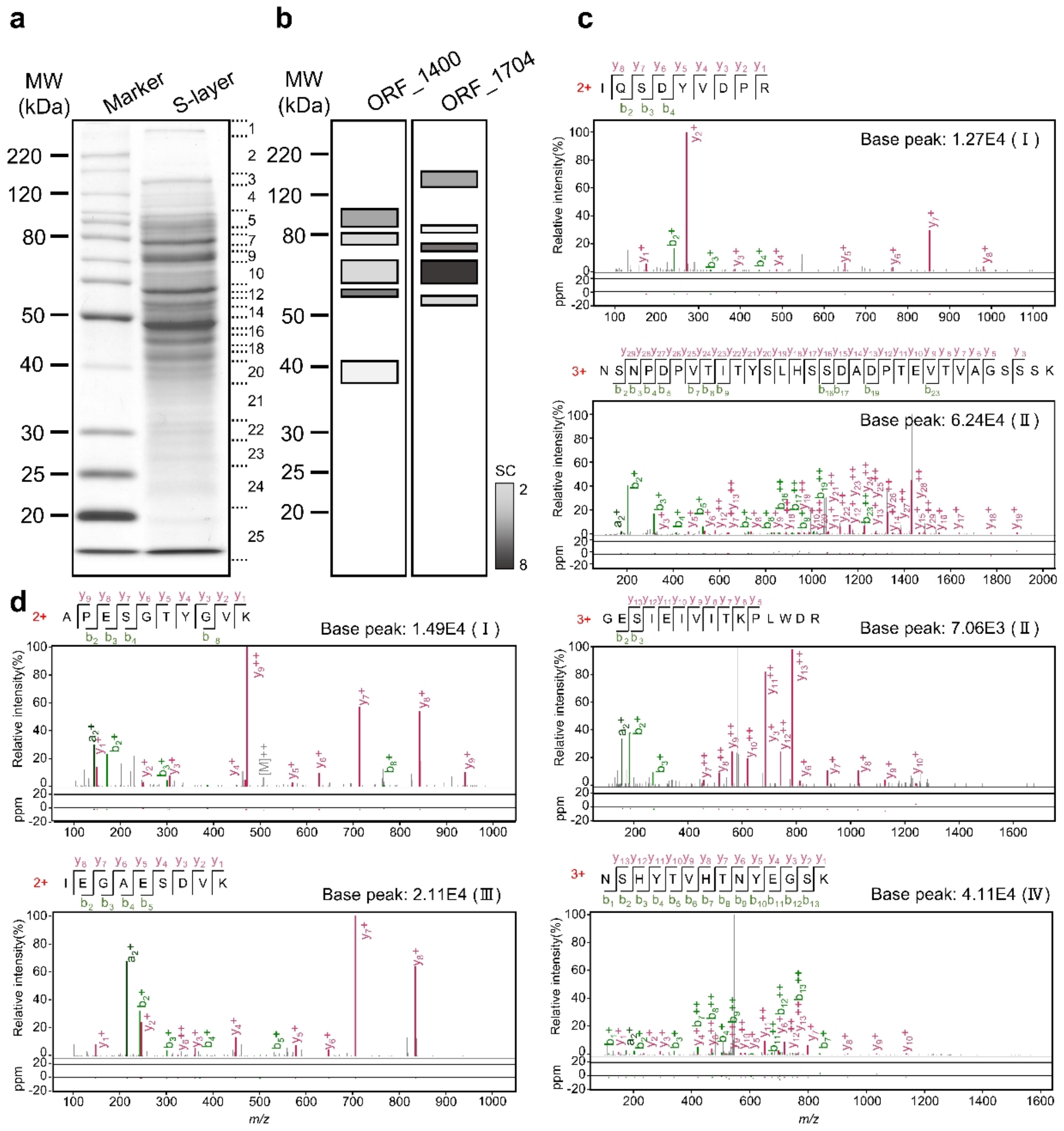

Supplementary Fig. 9 | Identification of S-layer proteins from YIM 93972 (n = 3). a, Separation of cell wall proteome by a 10% SDS-PAGE. **b**, The in silico reconstructed Western blots of the proteins ORF_1400 and ORF_1794, respectively. The color was used to represent the number and distribution of spectral counts for the identified S-layer proteins by LC-MS. The typical spectra of ORF_1400 (**c**) and ORF_1794 (**d**).

a

The selection of mutant colonies by the NTG mutagenesis.

Generation	Transitional	Bald	Total
1	513	597	1,110
2	174	129	303
3	23	230	253
4	5	3	8

b

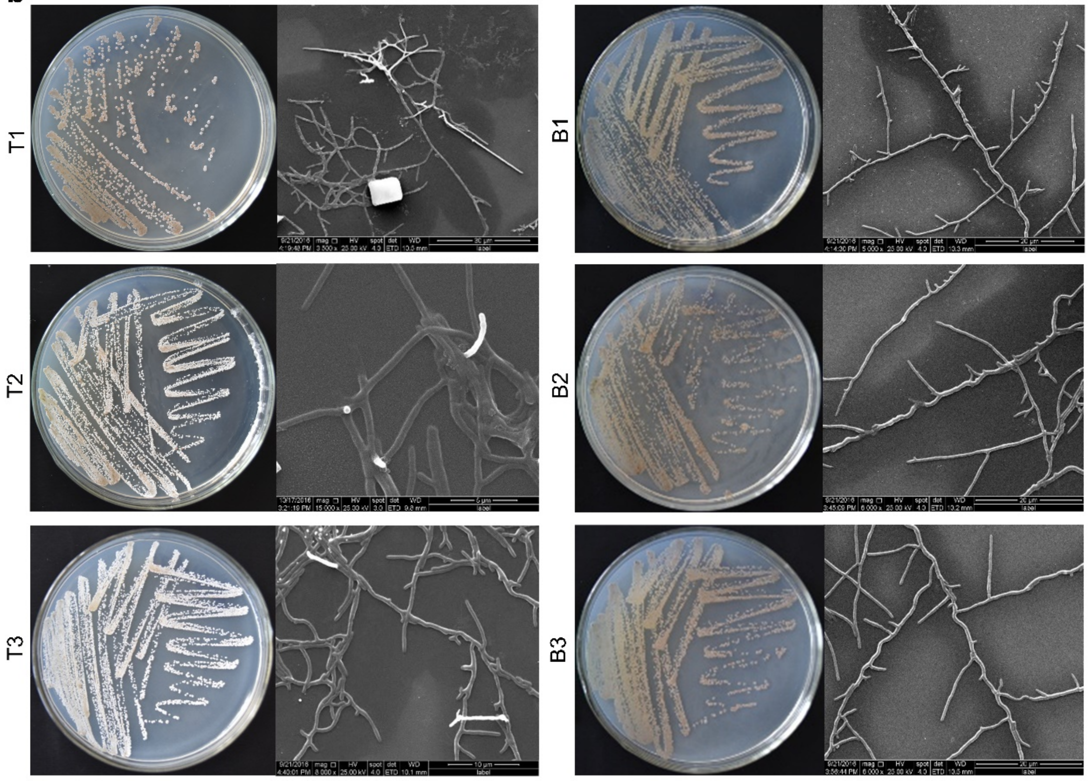

c The mutation analysis of five transitional and three bald mutated strains based on comparative genomics (No. of wild reads | No. of mutated reads).

Type	Strains	*ORF_0238* G1067A (Non-syn)	*ORF_0964* Syn	*ORF_2797* T-136G (Upstream)	*ORF_1717* C-69T (Upstream)
Wild	W1	248|1	249|1	248|1	248|0
	W2	247|0	250|0	247|0	249|0
Transitional	T1	248|0	249|0	250|0	248|0
	T2	245|2	248|0	248|0	1|238
	T3	245|1	248|0	247|2	247|0
	T4	249|1	248|0	248|0	0|242
	T5	247|0	249|0	249|1	0|241
Bald	B1	5|237	8|237	8|237	248|0
	B2	1|243	1|241	0|239	249|0
	B3	0|243	0|243	1|243	250|0

d The mutation analysis of two transitional and three bald mutated strains based on transcriptomics (No. of wild reads | No. of mutated reads).

Type	Strains	Biological duplication	*ORF_0238* G1067A (Non-syn)	*ORF_2797* T-136G (Upstream)
Wild	W1	W-AH1	58|0	0|0
		W-AH2	94|0	2|0
		W-AH3	5|0	0|0
		W-SH1	302|0	43|0
		W-SH2	332|0	103|0
		W-SH3	444|0	113|0
Transitional	T1	T1-SH1	0|329	0|93
		T1-SH2	0|300	0|118
		T1-SH3	46|647	14|174
	T2	T2-SH1	412|0	93|0
		T2-SH2	711|7	201|2
		T2-SH3	6|122	0|17
Bald	B1	B1-SH1	1|409	0|109
		B1-SH2	0|99	0|22
		B1-SH3	0|156	0|25
	B2	B2-SH1	2|447	2|100
		B2-SH2	0|366	0|120
		B2-SH3	0|210	0|45
	B3	B3-SH1	0|239	1|47
		B3-SH2	0|290	0|79
		B3-SH3	0|334	0|82

Supplementary Fig. 10 | The isolation and characteristics of YIM 93972 mutants. a, The numbers of mutant colonies of YIM 93972 by serial sub-cultivation. In each sub-cultivation, the morphological phenotypes of many mutants recovered to wild type. Therefore, after four generations, only 5 transitional mutants and 3 bald mutants remained with stable morphological mutant phenotypes. **b**, Scanning EM images of the mutants of strain YIM 93972 on solid ISP 4 medium (*n* = 3). T1, T2, and T3, three transitional colonies; B1, B2 and B3, three bald colonies. **c**, The single-base substitutions of the morphological mutants based on whole genome sequencing. **d**, The single-base substitutions of the morphological mutants based on RNA-seq. Abbreviations: W, wild type; T, transitional mutant; B, bald mutant; AH, aerial hyphae; SH, substrate hyphae.

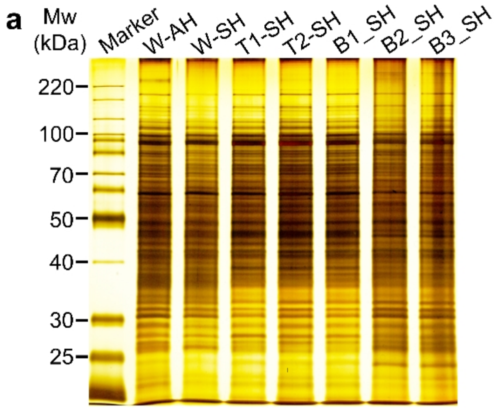

c

The strategy of sample combination.

Mixture	Fra	Fra	Fra	Fra	Fra
1	11	21	31	41	51
2	12	22	32	42	52
3	13	23	33	43	53
4	14	24	34	44	54
5	15	25	35	45	55
6	16	26	36	46	56
7	17	27	37	47	57
8	18	28	38	48	58
9	19	29	39	49	59
10	20	30	40	50	60

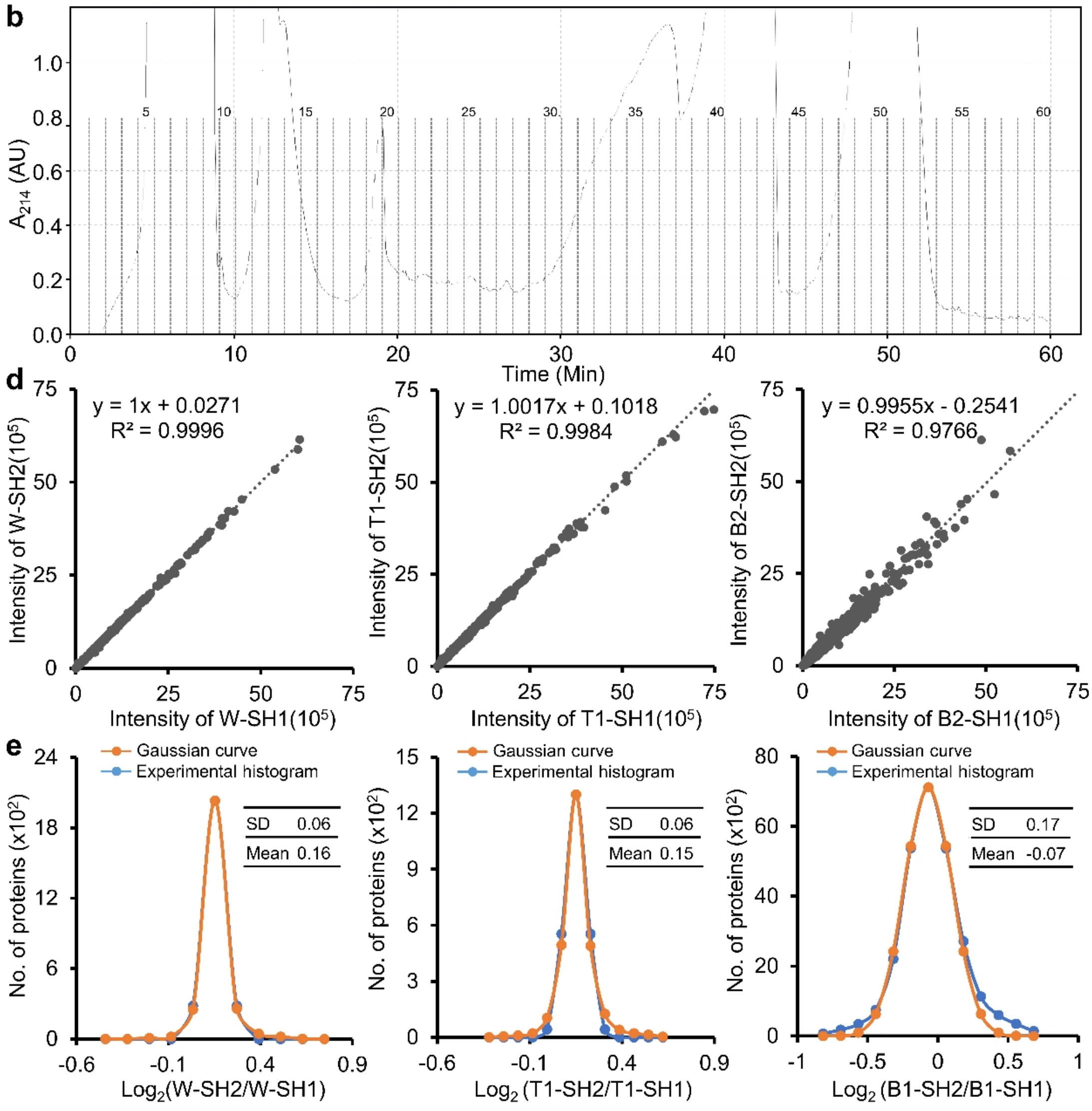

Supplementary Fig. 11 | The analysis of quantitative proteomics by TMT-labeling. a, 10% SDS-PAGE separation of strain YIM 93972 proteins from aerial and substrate hyphae samples of wild strain, substrate hyphae samples of transitional and bald strains. **b**, The RP HPLC chromatogram at AU_{214}. **c**, The combination of separated fractions. The

correlation (**d**) and gaussian fitting curve (**e**) of log_2 ratio of the intensities of two technical replicates in wild, transitional and bald group, respectively. The orange and blue curves represent the Gaussian fitting and experimental curve, respectively. Abbreviations: AH, aerial hyphae; SH, substrate hyphae; W, wild type; T, transitional mutant; B, bald mutant.

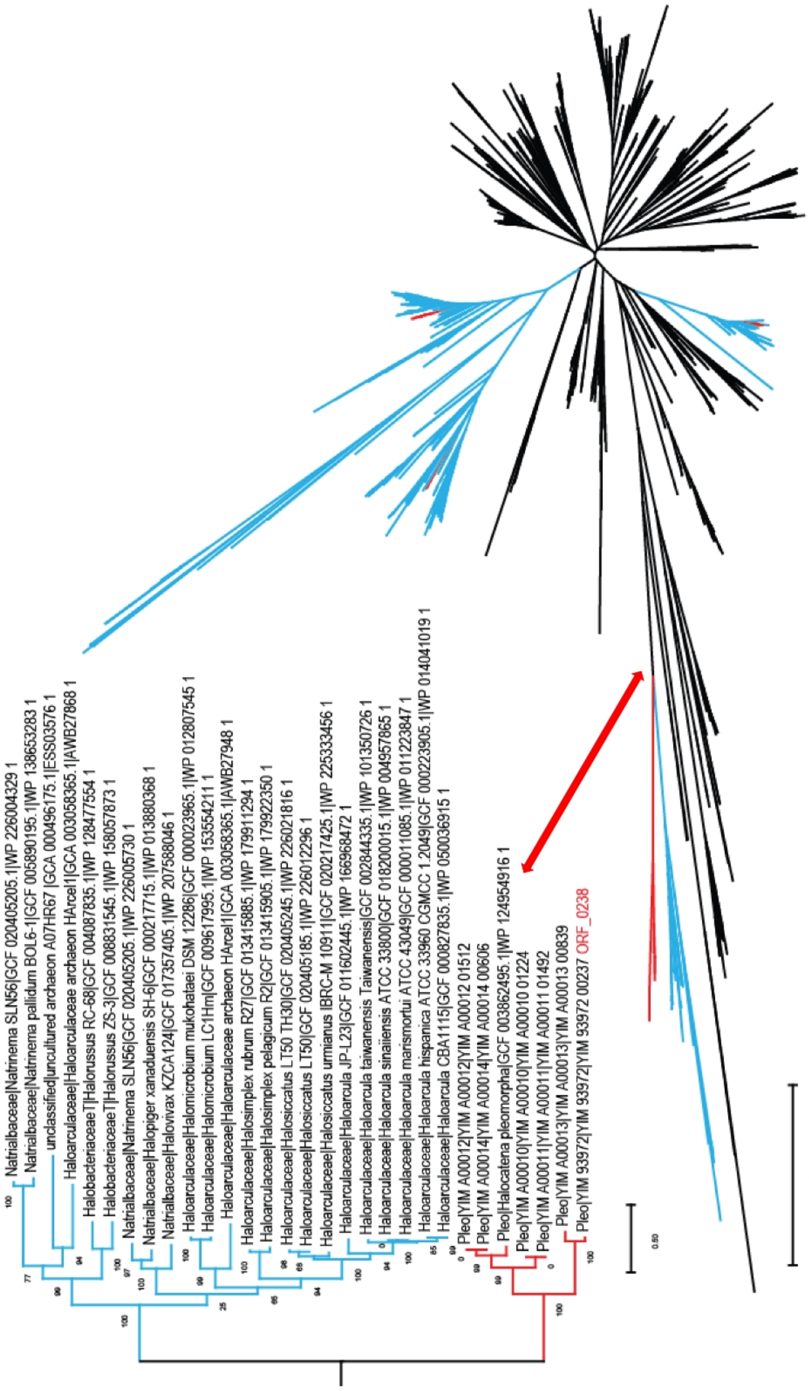

Supplementary Fig. 12 | Phylogenetic tree of the key gene *ORF_0238* (Cdc48 ATPase). The tree was built using two ATPase domains for all arCOG0308 members in archaea from arCOG database using FastTree program (WAG evolutionary model, gamma-distributed site rates); Blue – *Halobacteria*; Red – morphogenetic group; Black all other archaea.

Inhibition effect of bialaphos.

Concentration (μg/mL)	Wild	Transitional		Bald		
	W	T1	T2	B1	B2	B3
0.1	+	+	+	+	+	+
0.2	+	+	+	+	+	+
0.3	+	+	+	+	+	+
0.5	+	+	+	+	+	+
0.6	+	+	+	+	+	+
0.7	-	+	+	+	+	+
0.8	-	+	+	+	+	+
0.9	-	+	+	+	+	+
1.0	-	+	+	+	+	+

Supplementary Fig. 13 | The inhibition effect of bialaphos on wild, transitional and bald strains (*n*=3). Abbreviations: W, wild type; T, transitional mutant; B, bald mutant.

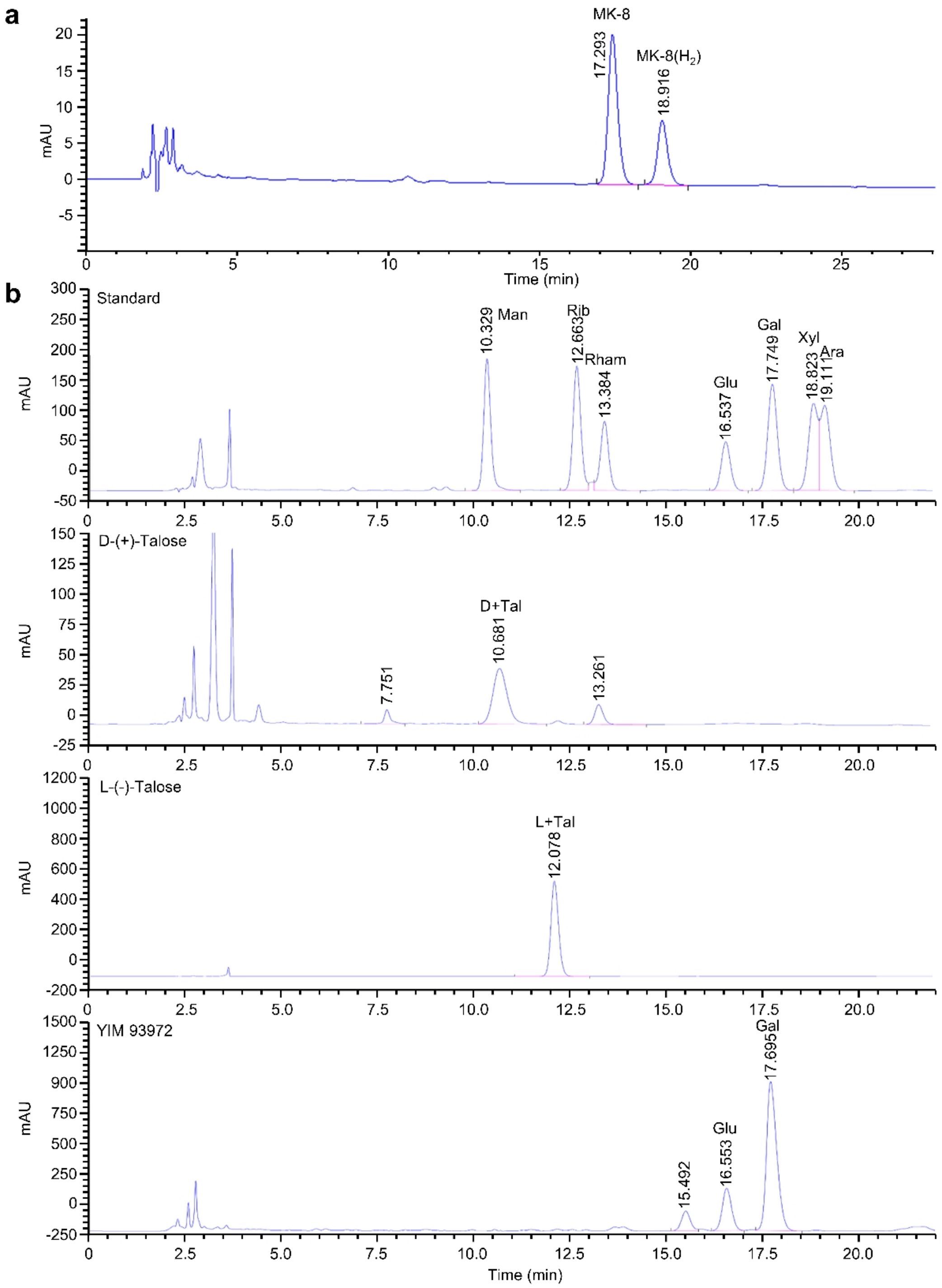

Supplementary Fig. 14 | The menaquinone and cell-wall sugar analysis of strain YIM 93972. a, The menaquinone analysis of strain YIM 93972 by HPLC. **b**, The cell-wall sugar components of strain YIM 93972 using HPLC based on the chromatograph of seven monosaccharide standards. Ara, Arabinose; Gal, galactose; Glc, glucose; Man, mannose; Rham, rhamnose; Rib, ribose; Tal, talose; Xyl, xylose.
