# Supplementary material for: Cellular differentiation into hyphae and spores in halophilic archaea
Source: Nat Commun. 2023 Apr 1;14:1827. doi: 10.1038/s41467-023-37389-w (PMC10067837; doi:10.1038/s41467-023-37389-w)
Supplement: Supplementary file 3 — Description of Additional Supplementary Files [file 41467_2023_37389_MOESM3_ESM.pdf]

Description of Additional Supplementary Files:

Supplementary Data 1. Differential characteristics of strain YIM 93972 and other type strains of reference species of the genera Halocatena, Halomarina and Natronomonas.

Supplementary Data 2. The annotation of six novel halobacteria with cellular differentiation.

Supplementary Data 2a. The genome annotation of strain YIM 93972.

Supplementary Data 2b. The genome annotation of strain YIM A00010.

Supplementary Data 2c. The genome annotation of strain YIM A00011.

Supplementary Data 2d. The genome annotation of strain YIM A00012.

Supplementary Data 2e. The genome annotation of strain YIM A00013.

Supplementary Data 2f. The genome annotation of strain YIM A00014.

Supplementary Data 3. 2270 common halo.COGs in morphogenetic halobacteria.

Supplementary Data 4a. 336 halo.COGs common in other Halobacteria but absent in morphogenetic halobacteria.

Supplementary Data 4b. Gains in the ancestor of morphogenetic group with probability >50% (according to GLOOME).

Supplementary Data 4c. Losses in the ancestor of morphogenetic group with probability >50% (according to GLOOME).

Supplementary Data 4d. Expansions in the core halo.COG set in morphogenetic halobacteria.

Supplementary Data 5. The single nucleotide polymorphisms (SNP) of DNA from wild (W), transitional (T) and bald (B) mutated strains based on Illumina Hiseq2500 sequencing (No. of wild reads | No. of mutated reads). T1-T5 are five biological replicates of transitional mutants. B1-B3 are three biological replicates of bald mutants.

Supplementary Data 6. Statistical table of the quality control data of transcriptomics (T_) by Illumina Hiseq sequencing. T1-T2 are two biological replicates of transitional mutants. B1-B3 are three biological replicates of bald mutants. Each sample has three technical replicates.

Supplementary Data 7. The SNP of DNA from wild (W), transitional (T) and bald (B) mutated strains at the transcriptome (T_) level. W-SH, wild substrate hyphae; W-AH, wild type aerial hyphae; T-SH, transitional substrate hyphae; B-SH, bald substrate hyphae. T1-T2 are two biological replicates of transitional mutants. B1-B3 are three biological replicates of bald mutants. Each sample has three technical replicates.

Supplementary Data 8. The quantification of total genes at the transcriptome (T_) level based on TPM value. W-SH, wild substrate hyphae; W-AH, wild type aerial hyphae; T-SH, transitional substrate hyphae; B-SH, bald substrate hyphae. T1-T2 are two biological replicates of transitional mutants. B1-B3 are three biological replicates of bald mutants. Each sample has three technical replicates.

Supplementary Data 9. Dysregulation analysis of total quantified genes at the transcriptome level. Statistical differences between two groups in transcriptomic datasets were analyzed using two-tailed unpaired t-tests (inter-group). $P < 0.05$ was considered statistically significant.W-SH was the control sample. Genes with ratios greater than 1.5-fold and p-value smaller than 0.05 (Student's t-test) were considered as regulated. W-SH, wild substrate hyphae; W-AH, wild type aerial hyphae; T-SH, transitional substrate hyphae; B-SH, bald substrate hyphae.

Supplementary Data 10. The protein identification at the proteome (P_) level. W-SH, wild substrate hyphae; W-AH, wild type aerial hyphae; T-SH, transitional substrate hyphae; B-SH, bald substrate hyphae. T1-T2 are two biological replicates of transitional mutants. B1-B3 are three biological replicates of bald mutants. W-SH1 and W-SH2, T1-SH1 and T1-SH2, B2-SH1 and B2-SH2, are two biological replicates from wild, transitional, and bald group, respectively.

Supplementary Data 11. The quantification of total 10-tag labeled proteins at the proteome level. W-SH, wild substrate hyphae; W-AH, wild type aerial hyphae; T-SH, transitional substrate hyphae; B-SH, bald substrate hyphae. T1-T2 are two biological replicates of transitional mutants. B1-B3 are three biological replicates of bald mutants. W-SH1 and W-SH2, T1-SH1 and T1-SH2, B2-SH1 and B2-SH2, are two biological replicates from wild, transitional, and bald group, respectively.

Supplementary Data 12. Dysregulation analysis of total quantified proteins at the proteome level. Statistical differences between two groups in proteomic datasets were analyzed using Significance A (intra-group). $P < 0.05$ was considered statistically significant. W-SH was the control sample. Protein changes greater than 1.5-fold and p-value smaller than 0.05 (Significance Aa) were considered as regulated. W-SH, wild substrate hyphae; W-AH, wild type aerial hyphae; T-SH, transitional substrate hyphae; B-SH, bald substrate hyphae. W-SH1 and W-SH2, T1-SH1 and T1-SH2, B2-SH1 and B2-SH2, are two biological replicates from wild, transitional, and bald group, respectively.

Supplementary Data 13. Dysregulation of orthologous groups (COG) classification of the differentially expressed genes and proteins. W-SH was the control sample. T_, dysregulated genes

from transcriptomics; P_, dysregulated proteins from proteomics. W-SH, wild substrate hyphae; W-AH, wild type aerial hyphae; T-SH, transitional substrate hyphae; B-SH, bald substrate hyphae.

Supplementary Data 14. Consistently regulated genes at the transcriptome (T_) and proteome (P_) level. W-SH was the control sample. W-SH, wild substrate hyphae; W-AH, wild type aerial hyphae; T-SH, transitional substrate hyphae; B-SH, bald substrate hyphae.

Supplementary Data 15. Strains and plasmids used in the verification of aerial mycelium differentiation controlling.

Supplementary Data 16. Oligonucleotide sequences used in this study.

Supplementary Data 17. The expression comparison of the potential transcriptional regulators predicted by DeepTFactor*.

Supplementary Data 18. Environmental SSU rRNA sequences of Supplementary Fig. 4a.

Supplementary Data 19. The expression clusters of total differentially expressed genes of Fig. 3d.

Supplementary Data 20. The expression clusters of total differentially expressed proteins Fig. 4e.

Supplementary Data 21. The gene expreesion comparision of the peptide ABC transporter in Fig. 5c.
