# Supplementary material for: Cellular differentiation into hyphae and spores in halophilic archaea
Source: Nat Commun. 2023 Apr 1;14:1827. doi: 10.1038/s41467-023-37389-w (PMC10067837; doi:10.1038/s41467-023-37389-w)
Supplement: Supplementary file 5 — Reporting Summary [file 41467_2023_37389_MOESM5_ESM.pdf]

nature portfolio

Corresponding author(s): Ping Xu, Guo-Ping Zhao, Eugene-V. Koonin, Shu-Kun Tang

Last updated by author(s): Feb 23, 2023

Reporting Summary

Nature Portfolio wishes to improve the reproducibility of the work that we publish. This form provides structure for consistency and transparency in reporting. For further information on Nature Portfolio policies, see our Editorial Policies and the Editorial Policy Checklist.

Statistics

For all statistical analyses, confirm that the following items are present in the figure legend, table legend, main text, or Methods section.

n/a	Confirmed	
☐	☒	The exact sample size (*n*) for each experimental group/condition, given as a discrete number and unit of measurement
☐	☒	A statement on whether measurements were taken from distinct samples or whether the same sample was measured repeatedly
☐	☒	The statistical test(s) used AND whether they are one- or two-sided<br>*Only common tests should be described solely by name; describe more complex techniques in the Methods section.*
☒	☐	A description of all covariates tested
☐	☒	A description of any assumptions or corrections, such as tests of normality and adjustment for multiple comparisons
☐	☒	A full description of the statistical parameters including central tendency (e.g. means) or other basic estimates (e.g. regression coefficient) AND variation (e.g. standard deviation) or associated estimates of uncertainty (e.g. confidence intervals)
☐	☒	For null hypothesis testing, the test statistic (e.g. *F*, *t*, *r*) with confidence intervals, effect sizes, degrees of freedom and *P* value noted<br>*Give P values as exact values whenever suitable.*
☒	☐	For Bayesian analysis, information on the choice of priors and Markov chain Monte Carlo settings
☐	☒	For hierarchical and complex designs, identification of the appropriate level for tests and full reporting of outcomes
☐	☒	Estimates of effect sizes (e.g. Cohen's *d*, Pearson's *r*), indicating how they were calculated

Our web collection on statistics for biologists contains articles on many of the points above.

Software and code

Policy information about availability of computer code

Data collection	The genome sequencing was performed on the Illumina novaseq 6000 platform. Environmental SSU rRNA and rRNA sequence was performed on the Illumina MiSeq PE300 platform. The mass spectrometry data were obtained on a Q Exactive HF mass spectrometer (ThermoFisher Scientific, Waltham, MA, USA) after Easy-Nano LC 1200 separation (ThermoFisher Scientific, Waltham, MA, USA).The RNA-seq was performed on an Illumina HiSeq 2000 platform.

Data analysis	For genomic sequencing, the Hierarchical Genome Assembly Process (HGAP, v2.3.0) pipeline was used to a generate high quality de novo assembly of the genome. The raw datasets were filtered, assembled by Canu (v1.5), and corrected by Pilon (v1.22). The rRNAs and tRNAs were predicted using RNAmmer (v1.2) and tRNAscan-SE (v1.3.1), respectively. The gene predication and annotation were performed using Prokka (v1.14.6).The circular maps were generated by DNAPlotter (v1.11).<br><br>For comparative genome and phylogenome, protein sequences were clustered using MMseqs2 (v14-7e284).Cluster alignments were obtained using MUSCLE (v5), and compared to each other using HHSEARCH (v3.3.0). The Maximum Likelihood phylogenetic tree (IQ-tree, v2.2.0, LG+F +R10 model) of Halobacteria was based on the BLOSUM62 alignment score and constructed using FastTree (v2) for the merged cluster alignments and rooted by mid-point. The history of gene gains and losses was reconstructed using GLOOME (v201305).<br><br>Database searching for the mass spectrometry raw data were performed using MaxQuant (v1.5.6.0); Statistical analyses (Student's t-test , Significance A, and spearman's correlation) and visualization were realized by R (v4.1.2). Cluster of the fold change based on the gene expression value at the mRNA and protein level were realized by R package Mfuzz (v2.58.0). The RNA-Seq reads per sample were mapped to the reference using Bowti2 (v 2.4.2). RSEM (v1.3.3) was used to estimate TPM. SNP analysis was performed by SAM tools (v1.12).

For manuscripts utilizing custom algorithms or software that are central to the research but not yet described in published literature, software must be made available to editors and reviewers. We strongly encourage code deposition in a community repository (e.g. GitHub). See the Nature Portfolio guidelines for submitting code & software for further information.

Data

Policy information about availability of data

All manuscripts must include a data availability statement. This statement should provide the following information, where applicable:

- Accession codes, unique identifiers, or web links for publicly available datasets
- A description of any restrictions on data availability
- For clinical datasets or third party data, please ensure that the statement adheres to our policy

The genome and transcriptome data generated in this study have been deposited in GenBank under accession codes CP071306-CP071310 and GSE168067, respectively. The raw reads of amplicon sequencing are deposited in GenBank, under accession number PRJNA703326. All MS data sets can be obtained from iProX71 database with identifier IPX0002724000 or ProteomeXchange with identifier PXD023481. Raw reads of genome for strains YIM_A00010, YIM_A00011, YIM_A00012, YIM_A00013, and YIM_A00014 were deposited in GenBank under accession codes JAKCFJ000000000, JAKCFK000000000, JAKCFL000000000, JAKCFM000000000, and JAKCFN000000000, respectively. Supplementary Data file 1 contains the complete Halobacterial COG data archive (supplementary_data_file_1.tgz), and supplementary data file 2 contains the phylogenetic trees and alignments archive (supplementary_data_file_2.tgz). They are located at https://ftp.ncbi.nih.gov/pub/wolf/_suppl/halo22/.

Human research participants

Policy information about studies involving human research participants and Sex and Gender in Research.

Reporting on sex and gender	The study did not involve human research.
Population characteristics	The study did not involve human research.
Recruitment	The study did not involve human research.
Ethics oversight	The study did not involve human research.

Note that full information on the approval of the study protocol must also be provided in the manuscript.

Field-specific reporting

Please select the one below that is the best fit for your research. If you are not sure, read the appropriate sections before making your selection.

[x] Life sciences [ ] Behavioural & social sciences [ ] Ecological, evolutionary & environmental sciences

For a reference copy of the document with all sections, see nature.com/documents/nr-reporting-summary-flat.pdf

Life sciences study design

All studies must disclose on these points even when the disclosure is negative.

Sample size	No statistical method was used to pre-determine the sample size. For the genomic, transcriptomic, and proteomic analysis of wild and mutated strains, the sample size was chosen similar to Streptomyces coelicolor project, while the statistical differences in the subtypes provide the rationale for sufficiency of the sample sizes. YIM 93972 and other five morphological differentiation strains were used to describe the morphological cellular differentiation in a haloarchaeon. For screening the morphological differentiation related genes, 2 poorly differentiated (transitional) and 3 undifferentiated (bald) mutants were used to perform transcriptomic and proteomic studies. For the transcriptomic experiment, three biological replicates were performed for each mutant.

Data exclusions	There is no exclusion in our data.
Replication	Two wild colonies of YIM 93972 were used for DNA extracting and genomic sequencing, respectively. For mutant selection, colonies were cultured and passaged for 4 times. For the transcriptomic experiment, three biological replicates were performed for each mutant. For the proteomic experiment, biological and technical replicates were performed. Totally 192 culture plates were prepared for each mutant, which were split into 6 groups as biological replicates. The substrate hyphae were recovered at 28 days. Totally, 6 pooled hyphae were collected for each strain, half of them were used for transcriptomic analysis, and another half were used for proteomic analysis.
Randomization	Samples were divided into three groups according to cellular morphological differentiation degree. Culture palates of each wild and mutant stain were were randomly divided into 6 pooled groups.
Blinding	The study did not involve blinding experiment.

Reporting for specific materials, systems and methods

We require information from authors about some types of materials, experimental systems and methods used in many studies. Here, indicate whether each material, system or method listed is relevant to your study. If you are not sure if a list item applies to your research, read the appropriate section before selecting a response.

Materials & experimental systems

n/a	Involved in the study
☒	☐ Antibodies
☒	☐ Eukaryotic cell lines
☒	☐ Palaeontology and archaeology
☒	☐ Animals and other organisms
☒	☐ Clinical data
☒	☐ Dual use research of concern

Methods

n/a	Involved in the study
☒	☐ ChIP-seq
☒	☐ Flow cytometry
☒	☐ MRI-based neuroimaging
